# Ferroptosis in hematological malignant tumors

**DOI:** 10.3389/fonc.2023.1127526

**Published:** 2023-04-17

**Authors:** Yong Liu, Zefan Du, Junbin Huang, Tianwen Li, Jing Zhang, Yixian Li, Wenfang Yi, Chun Chen

**Affiliations:** ^1^ Pediatric Hematology Laboratory, Division of Hematology/Oncology, Department of Pediatrics, The Seventh Affiliated Hospital of Sun Yat-Sen University, Shenzhen, Guangdong, China; ^2^ Department of Breast and Thyroid Surgery, Guangzhou Women and Children’s Medical Center, Guangzhou, Guangdong, China

**Keywords:** ferroptosis, ROS, GPx4, leukemia, lymphomas

## Abstract

Ferroptosis is a kind of iron-dependent programmed cell death discovered in recent years. Its main feature is the accumulation of lipid reactive oxygen species in cells, eventually leading to oxidative stress and cell death. It plays a pivotal role in normal physical conditions and the occurrence and development of various diseases. Studies have shown that tumor cells of the blood system, such as leukemia cells and lymphoma cells, are sensitive to the response to ferroptosis. Regulators that modulate the Ferroptosis pathway can accelerate or inhibit tumor disease progression. This article reviews the mechanism of ferroptosis and its research status in hematological malignancies. Understanding the mechanisms of ferroptosis could provide practical guidance for treating and preventing these dreaded diseases.

## Introduction

Ferroptosis is a novel iron-dependent mode of death induced by elastin and Ras selective lethal 3 (RSL3) (1). Different from apoptosis, classic necrosis, autophagy, and other forms of cell death. The morphological features of ferroptosis are reduced cell size, increased mitochondrial membrane density, and decreased cristae. It is characterized by the induction of lipid peroxides (LPO) accumulation dependent on iron ions and reactive oxygen species (ROS). Polyunsaturated fatty acid (PUFA) in phospholipids, REDOX active iron, and repair defects of LPO are three characteristics of ferroptosis, which determine the sensitivity of tumor cells to ferroptosis (2, 3). Targeted small molecule substances reduce the antioxidant glutathione (GSH) levels or glutathione peroxidase 4 (GPX4) by acting on specific targets in the cell, which leads to the accumulation of intracellular ROS and lipids. In the synergistic action of iron, peroxidation induces ferroptosis (4, 5). Cystine/glutamate antiporter (system Xc-) exists on the cell surface to maintain REDOX homeostasis, promote the entry of cystine into the cell, and then reduce it to cysteine, which removes excess glutamate from the cell and provides the raw material for intracellular GSH synthesis. Meanwhile, GSH is an essential substrate for the degradation of LPO by glutathione peroxidase 4 (GPX4). When the GSH synthesis pathway is inhibited, LPO accumulates, and ferroptosis occurs (6, 7). Ferroptosis requires GSH depletion, decreased glutathione peroxidase 4 (GPX4) activity, and the inability to metabolize lipid peroxides through GPX4-catalyzed glutathione reductase reaction, thereby destroying the integrity of the cell membrane (8). Initially, Dixon et al. suggested that ferroptosis is mainly regulated by ribosomal protein L8 (RPL8), iron response element binding protein 2 (IREB2) at the gene level. ATP synthase F0 complex subunit C3 (ATP5G3), tetratricopeptide repeat domain 35 (TTC35), Regulation of citrate synthase (CS) and acyl-CoAsynthetase family member 2 (ACSF2) (1). Later, many studies have shown that many genes/proteins and molecular are involved in this particular cell death process, such as cyclooxygenase 2(PTGS2) (9), p53 (10), nuclear factor E2-related factor 2(Nrf2) (11), phosphatidylethanolamine binding protein 1(PEBP1) (12) and miRNA (13). In addition to these key regulators, the onset of ferroptosis is associated with excess glutamate, an increase in the intracellular iron concentration, or the handling of small-molecule substances such as elastin, RSL3, and others in **Table 1**.

**Table 1:** Main inducers, inhibitors, and regulators in hematological malignant tumors relevant to ferroptosis.

Target	Compound name/protein	Effect	Hematological Malignant Tumors with Relevance to Ferroptosis
Inducers			
VDACs and system $x_{c}^{-}$	Erastin Piperazine erastin	Induce ferroptosis	Chronic Myelogenous Leukemia [14] Acute myeloid leukemia [9] Diffuse large B cell ymphomas[9]
GPX4	RSL3	Induce ferroptosis	Acute myeloid leukemia [9] Diffuse large B cell lymphomas [9]
System $x_{c}^{-}$	Sulfasalazine	Induce ferroptosis	Non-Hodgkin’s lymphoma [15]
Lipid peroxidation	FINO2	Induce ferroptosis	B-lymphoblastic cell leukemia[16] B-cell lymphoma[16]
GSH depletion	Buthioninesulfoximine	Induce ferroptosis	Acute myeloid leukemia [9] Diffuse large B cell lymphomas [9]
Inhibitors			
P38	SB202190	Inhibit ferroptosis	Acute myeloid leukemia[17]
ROS from lipid peroxidation	Ferrostatin-1	Inhibit ferroptosis	Acute lymphoblastic leukemia [18]
Intracellular iron	Ciclopirox olamine	Inhibit ferroptosis	Acute lymphocytic leukemia [19]

In recent years, with the in-depth study of the mechanism of ferroptosis, ferroptosis has been found in a variety of tumor cells, including breast cancer (20), lung cancer (1), liver cancer (21), kidney cancer (22) and brain tumors (23). In 2015, Jiang et al. found that p53 is essential in regulating ferroptosis, and this study found that ferroptosis contributes to embryonic development (10). Leukemia and lymphoma are the most common hematological malignancies. The main treatment methods are chemotherapy and stem cell transplantation. Although the treatment level of stem cell transplantation has been dramatically improved in recent years, its application has certain limitations (24). At the same time, the remission rate of chemotherapy is low, and there has been no substantial progress in recent years. Therefore, exploring treatment options that will benefit patients is still necessary. As one of the cell death modes, ferroptosis is a popular research direction in tumor research and treatment (25, 26). The known ferroptosis inducers can be divided into the following two categories. The first category includes Erastin, sulfasalazine, glutamate. Which can act through system xc-. The second class includes RSL3 and DP17, which can directly inhibit glutathione peroxidase activity (GPX). Unlike other ferroptosis inducers that usually mediate a single pathway, Erastin can mediate multiple molecules with efficient, rapid, and long-lasting effects (5, 27). In addition, depending on the mechanism of ferroptosis, many molecules have been identified as ferroptosis inhibitors, including ferrostatin-1(Fer-1, inhibiting lipid ROS) (1), deferoxamine (DFO, chelating iron) (1). Examples include deferoxamine (DFO, chelating iron) (1), zileuton (inhibiting 5-lipoxygenase) (28), and the newly discovered FINO2(iron oxide) (14). This article reviews the research progress of ferroptosis in hematological malignancies, and the main inducers, regulators, and inhibitors of ferroptosis in hematological malignancies, as shown in **Table 1**.

## Iron metabolism and hematological tumors

Iron is a nutrient that promotes cell metabolism, proliferation, and growth. Iron ions can gain and lose electrons. They are the active sites of many iron- and heme-containing enzymes in the body, such as mitochondrial enzymes related to respiratory complexes, enzymes related to DNA synthesis and cell cycle, and enzymes related to detoxification functions (such as peroxidase and catalase); the ability to freely gain and lose electrons allows iron to participate in redox reactions and form free radicals (15). Through the Fenton reaction, hydrogen peroxide reacts under the catalysis of ferrous iron to generate reactive oxygen species (16) Reactive oxygen species can causelipid and protein damage and oxidative DNA damage, including DNA base modification and DNA strand breaks, inducing mutations and promoting the occurrence and progression of tumors (17). In recent years, evidence has shown that ROS are essential for health. Under physiological conditions, generating low levels of ROS is considered a signaling molecule. On the other hand, abnormal iron accumulation, ROS overproduction, and ROS buffer system dysfunction can cause many chronic diseases. Therefore, the chronic accumulation of ROS is involved in many diseases. Moreover, excessive ROS production induces oxidative damage of biomolecules, leading to aging, cancer, and many other diseases (18). Many studies have shown that iron metabolism disorders are related to the occurrence of a variety of diseases, including hereditary hemochromatosis, myocardial ischemia-reperfusion injury, neurodegenerative diseases, and even cancer (19, 29, 30). Specifically, excessive intracellular iron levels can mediate carcinogenesis due to its pro-oxidative properties and DNA-damaging effects. At the same time, tumor cells acquire large amounts of iron to maintain rapid growth and proliferation (31). Under normal homeostasis, transferrin binds to free iron in the circulation, and metabolic iron in the body is maintained at a steady state. When iron overload occurs in the body, serum transferrin binding is close to saturation, and there is non-transferrin-bound iron (NTBI) in circulation (6, 32, 33). However, excessive iron can increase intracellular ROS content through the Fenton reaction, thus promoting ferroptosis (16). *In vitro* treatment of mouse primary hepatocytes and bone marrow-derived macrophages (BMDMs) with ferric ammonium citrate (FAC) significantly increased lipid peroxidation, decreased NADPH, and decreased cellular viability. These changes were reversed by ferroptosis inhibitors and iron chelators (34). In addition, the team also found that the Slc7a11-/- mouse model did not cause ferroptosis under basal iron conditions. The high-iron diet caused increased non-transferrin-bound iron in mice, decreased GSH levels, and increased ROS levels, indicating that iron-induced ferroptosis differs from that induced by erastin. Nuclear receptor coactivator 4 (NCOA4) is a selective receptor for the selective autophagy flip of ferritin in ferroptosis Hou et al. knocked out NCOA4 in PANC1 cells, which reduced the level of intracellular divalent iron. Ferroptosis is induced by erastin, and the level of iron is increased in the cells overexpressing NCOA4 by transfection, and the level of ferroptosis is increased. Therefore, the increase of intracellular iron caused by the degradation of ferritin mediated by NCOA4 participates in ferroptosis (35). The exosomes secreted by GPX4 inhibitor-treated cells contain a large amount of ferritin (36).

**Figure 1:**
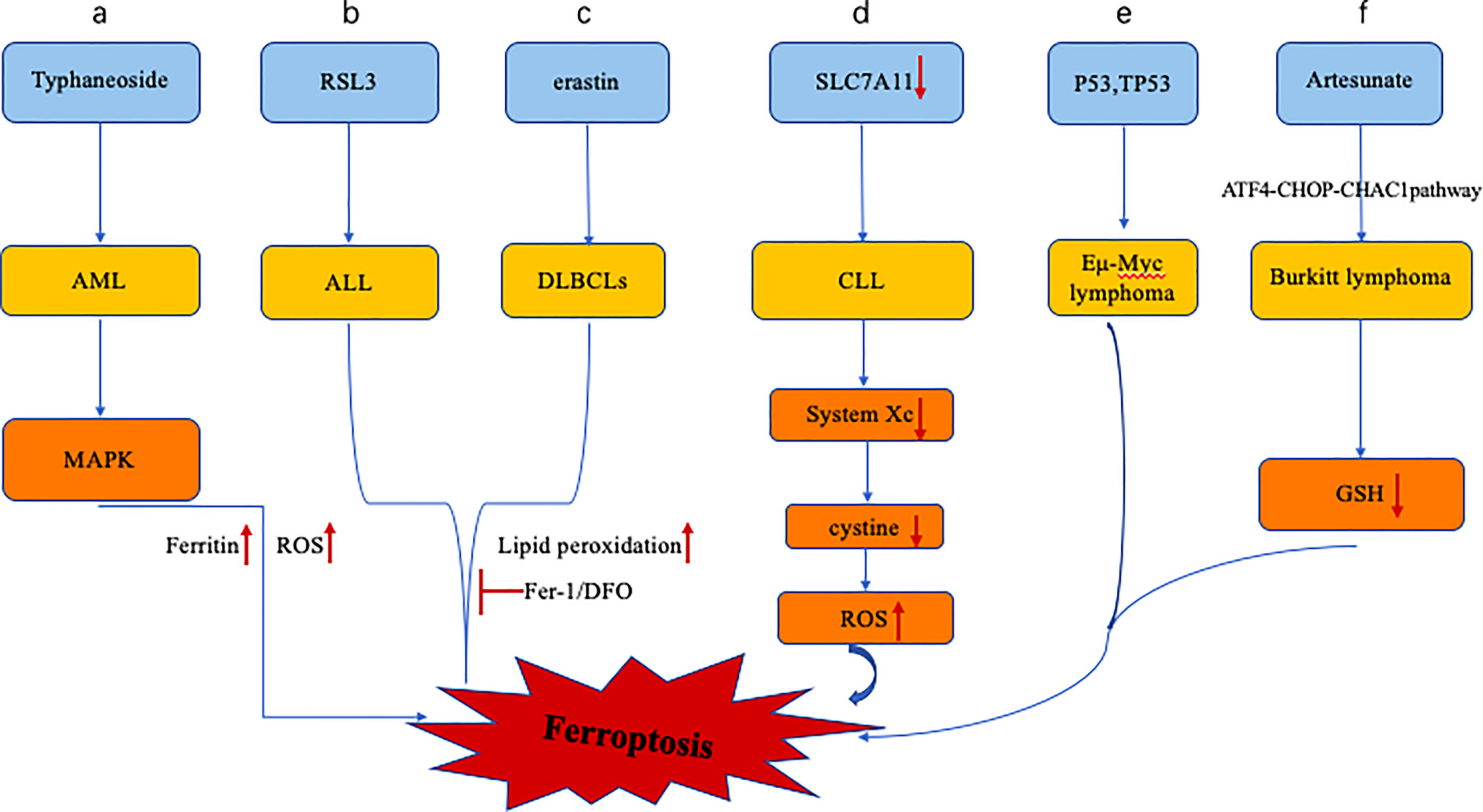
Altered iron metabolism in leukemia includes dysregulation of the ferroportin-hepcidin regulatory axis. **(A)** Typhaneoside treatment of AML cells resulted in ferroptosis in AML cells by activating AMPK signaling, accompanied by ferritin degradation and ROS accumulation. **(B, C)** RSL3 and erastin treatment of ALL and DLBCLs resulted in ferroptosis, accompanied by increased lipid peroxidation, which was inhibited by antioxidant and DFO. **(D)**, Expression of SLC7A11 in CLL cells was down-regulated, and the system xc- transporter cystine ability was decreased, leading to the increase of intracellular ROS and the promotion of cell ferroptosis. **(E)**, p53 inhibits System xc- and promotes ferroptosis in Eμ-Myc lymphoma cells, while the deletion of TP53 gene accelerates the formation of Eμ-Myc lymphoma model. **(F)**, Artesunate can induce ferroptosis in Burkitt lymphoma cells by activating ATF4-CHOP-CHAC1 pathway and degrading GSH.

Compared with normal hematopoietic cells, leukemic cells have altered iron uptake, storage, and efflux and an altered iron transporter-hepcidin regulatory axis (**Figure 1**) (17). Although iron and the reactive oxygen species generated by its catalysis are crucial to maintaining the balance of the hematopoietic system, the accumulation of iron and the subsequent abnormal increase in reactive oxygen species can disrupt various biological functions of hematopoietic stem cells (HSCs) (37), including quiescence, self-renewal, and multilineage differentiation potential. Excessive ROS promotes cell senescence and apoptosis and impairs self-renewal ability and tumor formation, similarly, too much iron can lead to changes in the tumor microenvironment that promote cancer cell ferroptosis (38, 39). In addition, iron is crucial to the development of leukemia because iron-dependent ribonucleotide reductase is required for DNA synthesis to maintain the rapid proliferation of leukemia cells (40–42). In addition, iron overload induces apoptosis of neighboring NK cells, CD4+ T cells, and CD8+ T cells while simultaneously increasing the ratio of regulatory T cells, allowing leukemia cells to evade immune system attack (43, 44). In addition, unlike malignant tumors of other systems, patients with hematological malignancies require repeated blood transfusions due to normal erythropoiesis and chemotherapy, resulting in increased iron load in the body. Excessive iron and ROS can promote the malignant transformation of hematopoietic stem cells by consuming smoke NADPH oxidase (NOX) and GSH (39). In myelodysplastic syndromes, ROS-induced DNA damage may increase patients’ risk of leukemia (38). These results indicate that excessive iron promotes ferroptosis through the ROS pathway, and changes in intracellular iron levels mediated by the ferritin metabolism pathway are also closely related to ferroptosis. Regulating the homeostasis of iron metabolism alters the susceptibility of AML cells to ferroptosis, and disease progression in leukemia also increases iron accumulation in patients.

## The mechanism of ferroptosis and its research in hematological tumors

### ROS

Reactive oxygen species (ROS), produced during normal metabolic processes, play essential roles in cell signaling and tissue homeostasis. However, ROS overproduced during metabolic processes are key promoters of the ferroptosis cascade and can lead to unfavorable modifications of various intracellular molecules such as lipids, proteins, and DNA damage (45, 46). This is called “lipid peroxidation.” It is generally believed that lipid peroxides, especially the peroxidation of lipid hydrogen, can lead to damage reactions in the lipid bilayer of the cell membrane, which is a signal of cell death and can induce programmed cell death is an essential mediator of ferroptosis (47). Besides, lipid peroxides can produce additional toxicity due to their degradation products by altering the structure and function of nucleic acids and proteins such as Michael receptors and aldehydes (48). The high-throughput screening results showed that ferstatin-1 (fer-1) and lipoxstatin-1 (lip-1) could prevent erastin-induced ROS accumulation, specifically inhibiting RSL-induced ferroptosis. This also demonstrates the role of ROS accumulation in ferroptosis (49).

### GPX4

GPX4 is one of the 25 human genome proteins containing selenocysteine and belongs to the GPxs family (24). It uses GSH as a cofactor to reduce lipid peroxide to lipid alcohol, preventing ROS synthesis (9, 50). GPX4 is the direct target of RSL3. GPX4 can avoid the toxicity of lipid peroxides and maintain the stability of the membrane lipid bilayer through its enzymatic activity (51). RSL3 can specifically inhibit the activity of GPX4, which leads to ROS accumulation in cells and induce ferroptosis (52). Furthermore, overexpression of GPX4 can reduce ferroptosis (27). Pedro et al. Demonstrated that the knockout of the GPX4 gene resulted in ferroptosis in cells (53). GPX4 ‐ deficient mouse embryonic fibroblasts showed resistance to erastin-induced cell death, suggesting that ferroptosis depends on ROS (54). Selenium can effectively inhibit GPX4-dependent ferroptosis by activating tfap2c and Sp1 to enhance genes in this transcription program, such as GPX4. Selenium deficiency can inactivate GPX4, thus making cells more sensitive to oxidative damage (55, 56). In addition, fino2 and fin56 induced ferroptosis by indirectly inhibiting GPX4 levels and activity without interfering with glutathione metabolism (57, 58).

### System Xc−

Glutamate‐cystine antiport system x c – (system x c −) is a component of cell membrane transporter and is a heterodimer composed of SLC7A11 and SLC3A2, and responsible for the exchange of extracellular cystine and intracellular glutamate (58). Under physiological conditions, extracellular cystine is transported into the cell through system Xc-, the substrate for synthesizing the antioxidant glutathione, and glutathione is the main component for removing reactive oxygen species (59). Blocking system xc - can inhibit the synthesis of cysteine-dependent glutathione (GSH) and then damage cells’ antioxidant defense ability, thereby promoting ROS accumulation and inducing ferroptosis in cells. For example, sulfasalazine can inhibit system xc - and cause ferroptosis, while β-mercaptoethanol increases cystine uptake, thus inhibiting elastin-induced ferroptosis in HT1080 cells (5). Erastin and sulfasalazine prevented cultured cancer cells from absorbing radiolabeled cystine (60).

Using traditional biochemical methods and more advanced metabonomics analysis, erastin treatment can lead to large consumption of intracellular GSH (61). How erastin or SAS inhibits SLC7A11-mediated Cys2 import remains unclear. Initial studies suggested that erastin could bind to the related transporter SLC7A5 and further inhibit SLC7A11 in trans2. However, recent findings deny this possibility and suggest that erastin can directly inhibit SLC7A11 (60). Previous studies have revealed more potent drug analogs of erastin, and these findings are helpful for future studies on the targets and effects of erastin *in vitro* and *in vivo* (62). Wang et al. found that the source of cystine/cysteine in SLC7A11 knockout mice was decreased, which limited the subsequent GSH synthesis and increased the sensitivity of cells to ferroptosis induced by iron overload (34). Similarly, studies have shown that Nrf2 plays a key role in leukemia cell resistance to DOX and in triptolide induced iron death, suggesting a potential strategy for using triptolide and DOX in combination therapy in leukemia treatment (63). Moreover, Sorafenib induces ferroptosis in hepatocellular carcinoma through SHP-1/Stat3-mediated MCL1 downregulation and subsequent inhibition of SLC7A11 by increased BECN1 binding (64).

In summary, the accumulation of ROS and the increase of lipid peroxidation are associated with ferroptosis. The level and activity of GPX4 can affect the level of ferroptosis. In addition, system xc - regulates ROS balance by affecting GSH metabolism, an essential part of ferroptosis.

Jin et al. found that dihydroartemisinin (DHA) can strongly inhibit the activity of AML cell lines and can further effectively induce the death of AML cells, which is often considered ferroptosis because of its apparent iron dependence and accompanied by mitochondrial dysfunction. Mechanistically, DHA induces autophagy by regulating the AMPK/mTOR/p70S6k signaling pathway, which can accelerate ferritin degradation and increase the labile iron pool, further promoting the accumulation of ROS in cells and eventually leading to ferroptosis (65). Probst et al. Used acute lymphoblastic leukemia (all) cell lines as a cell model. Increased lipid peroxidation levels accompanied cell death after rsl3 treatment. The cell death was inhibited by adding a lipid peroxidation inhibitor fer-1 or lipoxygenase (LOX), and the iron chelator DFO could reverse RSL3-triggered cell death (66). Data analysis showed that diffuse large B lymphomas (DLBCLs) were particularly sensitive. Besides, lipid peroxides are produced in the DLBCL cell line treated with erastin. Lipophilic antioxidants can save cell death, indicating that the cell death in this cell line has ferroptosis characteristics. The sensitivity of DLBCL cell lines and other hematopoietic cell lines to 203 different lethal compounds was further analyzed, and it was found that the average resistance of DLBCL cell lines to all compounds was weak, indicating that the enhanced sensitivity of DLBCLs to erastin-induced ferroptosis was not due to Universal sensitivity to all compounds (9).

These results suggest that the susceptibility of leukemia and lymphoma cells to ferroptosis is consistent with the response to classical ferroptosis, which is characterized by excessive accumulation of ROS and increased lipid peroxidation. Typhaneoside (TYP) is a major flavonoid in the extract of Pollen Typhae, showing significant biological and pharmacological effects. Typ activates the AMP-activated protein kinase (AMPK) signaling pathway, which leads to ferritin degradation, ROS accumulation, and ferroptosis, and further significantly triggers autophagy in AML cells (67). Similarly, artesunate can induce an ER stress response and activate the ATF4-CHOPCHAC1 pathway to degrade intracellular GSH, thereby inducing ferroptosis in Burkitt lymphoma cells. The protection of the cells evidences this by Lip-1, Fer-1, and DFO (68). These studies provide new ideas for promoting ferroptosis in treating hematological malignancies. Human lymphoma and leukemia cells cannot convert methionine to cystine by their metabolic functions. Therefore, its growth and proliferation must be mediated by the extracellular uptake of cysteine. Interestingly, SLC7A11 was down-regulated in chronic lymphocytic leukemia (CLL) compared with other systemic solid tumors. The systemic xc transporting cystine capacity was reduced, which could increase the intracellular ROS level. This suggests that CLL is strongly associated with ferroptosis (69). Similarly, clinical studies have also shown that in patients with DLBCLs, GPX4 expression accounted for 35.5%(33/93). The survival time of the gpx4 positive group was significantly higher than that of the gpx4 negative group (70). GPX4 overexpression is an independent prognostic indicator of diffuse large B-cell lymphoma and AML (71, 72). This may be related to the role of GPX4 in reducing intracellular lipid oxidation and making cells insensitive to ferroptosis. In conclusion, ROS accumulation and lipid peroxidation play essential roles in the mechanism of ferroptosis. Studies on ferroptosis sensitivity show that leukemia and lymphoma cells can be more sensitive to ferroptosis by increasing the accumulation of intracellular ROS through multi-channel regulation. This opens up a new research direction for selecting drugs for the treating hematological tumors.

### P53 and hematological tumors

p53 controls the cell cycle, DNA replication, and uncontrolled cell division during tumor growth. When p53 is mutated, it leads to tumor initiation and progression (71). By inhibiting the transcription of SLC7A11, p53cystine uptake, reduces and intracellular GSH, increases intracellular ROS accumulation, and increases the susceptibility to cell ferroptosis. They used an acetylation-deficient mutant p53 (3KR) that cannot induce other forms of apoptosis but retains the ability to regulate the expression of SLC7A11 and found that SLC7A11 is overexpressed in many types of human cancers. High expression levels of SLC7A11 can significantly reduce the tumor growth inhibitory activity induced by p53(3KR), indicating that this inhibitory activity has nothing to do with the disturbance of cell cycle, cell apoptosis, and cell senescence. At the same time, high levels of reactive oxygen species can trigger p53-mediated ferroptosis. The regulation of ROS levels by p53 is a complex biological process. When ROS levels are at basal or relatively low levels, p53 can prevent cellular cells from continuing to accumulate ROS. However, when ROS levels are abnormally high, p53 may promote cell death through the ferroptosis pathway. Therefore, ferroptosis can be regulated by p53 affecting intracellular ROS levels. The Alox12 gene is located on human chromosome 17p13.1 in a very close position to the TP53 gene. Some scholars have suggested that many human tumors lose one alox12 allele (73). Chu et al. used p53(3KR) H1299 cells lacking six lipoxygenase isoforms to test ROS-induced ferroptosis levels after butyl alcohol (TBH) treatment and found that p53-mediated ferroptosis could be specifically blocked by loss of ALOX12 function. SLC7A11 specifically binds to alox12, thereby reducing its enzymatic activity, confirming that p53 can indirectly activate ALOX12 lipoxygenase activity by inhibiting SLC7A11 transcriptional repression system Xc, leading to Alox12-dependent ferroptosis induced by ROS death. The pathway of ferroptosis is independent of the GPX4 pathway (74).

Thus, p53 regulates ferroptosis levels by regulating SLC7A11 transcript levels and activity. Further studies showed that alox12 deletion inhibited p53-mediated iron mineralization. In the Eμ-myc mouse model, the loss of one Trp53 allele significantly accelerated the formation of the Myc-induced classical eμ-myc lymphoma model. In contrast, the loss of an alox12 allele shortened the median survival of these mice (74). In conclusion, p53 increases the sensitivity of cell death with iron by regulating ROS level, and the loss of p53 function plays an essential role in the occurrence and prognosis of Eμ-myc lymphoma.

## Conclusion

Ferroptosis is a cell death mediated by various small molecules, such as erastin and RSL3. It is affected by GPX4, GSH metabolism, iron metabolism, and other pathways. Its occurrence is often accompanied by the accumulation of ROS and other substances, which can further lead to the peroxidation of cell membrane lipids. The specific mechanism of ferroptosis needs further study. The study of cell death mode is still an essential link in overcoming the tumor treatment problem. In recent years, ferroptosis has been more popular in tumor research. Many studies have shown that the sensitivity of leukemia and lymphoma cells to ferroptosis can be regulated by changing the concentration of ferroptosis-inducing molecules, the balance between intracellular ROS level and cell death, and the level of intracellular iron metabolism, to achieve the goal of eliminating leukemia and lymphoma cells. Studies have also found that many compounds are closely related to the ferroptosis of tumor cells, and the level of ferroptosis inducers is related to the prognosis of the disease. In addition to the existing iron chelators and targeted iron metabolism-related proteins, treating redox imbalance disorders targeting high intracellular iron levels also has broad application prospects in treating leukemia. The application of ferroptosis and ferromacrophages as a new ferroptosis therapy in leukemia has just begun. With the rapid development of nanotechnology, efforts have been made to exploit the therapeutic advantages of iron-based nanoparticles. The Magnetic field can not only concentrate nanoparticles but also promote the production of intracellular ROS, thus exerting an anti-leukemia effect. The progression of blood system tumor diseases and the changes brought about by treatment will also affect the ferroptosis process. There will be expected to be more related studies to provide new ideas for treating leukemia and lymphoma.

## Author contributions

(I) Conception and design: CC, YLiu, ZD. (II) Administrative support: None. (III) Provision of study materials or patients: YLiu, ZD, JH, TL, JZ, WY, YLi. (IV) All authors performed data collection and summary. (V) Data analysis and interpretation: Not applicable. (VI) Manuscript writing: All authors. All authors contributed to the article and approved the submitted version.
